# Assessment of faculty productivity in academic departments of medicine in the United States: a national survey

**DOI:** 10.1186/1472-6920-14-205

**Published:** 2014-09-26

**Authors:** Victor F Kairouz, Dany Raad, John Fudyma, Anne B Curtis, Holger J Schünemann, Elie A Akl

**Affiliations:** Department of Medicine, State University of New York at Buffalo, Buffalo, New York, USA; Department of Clinical Epidemiology and Biostatistics, McMaster University, Hamilton, Ontario Canada; Department of Medicine, McMaster University, Hamilton, Ontario Canada; Department of Internal Medicine, American University of Beirut, Riad-El-Solh, P.O. Box: 11-0236, Beirut, 1107 2020 Lebanon

**Keywords:** Faculty productivity, Salary compensation, Academia, Department of medicine, Survey

## Abstract

**Background:**

Faculty productivity is essential for academic medical centers striving to achieve excellence and national recognition. The objective of this study was to evaluate whether and how academic Departments of Medicine in the United States measure faculty productivity for the purpose of salary compensation.

**Methods:**

We surveyed the Chairs of academic Departments of Medicine in the United States in 2012. We sent a paper-based questionnaire along with a personalized invitation letter by postal mail. For non-responders, we sent reminder letters, then called them and faxed them the questionnaire. The questionnaire included 8 questions with 23 tabulated close-ended items about the types of productivity measured (clinical, research, teaching, administrative) and the measurement strategies used. We conducted descriptive analyses.

**Results:**

Chairs of 78 of 152 eligible departments responded to the survey (51% response rate). Overall, 82% of respondents reported measuring at least one type of faculty productivity for the purpose of salary compensation. Amongst those measuring faculty productivity, types measured were: clinical (98%), research (61%), teaching (62%), and administrative (64%). Percentages of respondents who reported the use of standardized measurements units (e.g., Relative Value Units (RVUs)) varied from 17% for administrative productivity to 95% for research productivity. Departments reported a wide variation of what exact activities are measured and how they are monetarily compensated. Most compensation plans take into account academic rank (77%). The majority of compensation plans are in the form of a bonus on top of a fixed salary (66%) and/or an adjustment of salary based on previous period productivity (55%).

**Conclusion:**

Our survey suggests that most academic Departments of Medicine in the United States measure faculty productivity and convert it into standardized units for the purpose of salary compensation. The exact activities that are measured and how they are monetarily compensated varied substantially across departments.

**Electronic supplementary material:**

The online version of this article (doi:10.1186/1472-6920-14-205) contains supplementary material, which is available to authorized users.

## Background

Academic medical centers have become a large unit where clinical and research success are synergistic [1] and clinical revenue is strategically aligned with academic performance[2], leading to a collaboration between hospital and university leaders to enhance and improve academic productivity. Faculty productivity can be defined as a measurable output of a faculty member related to clinical, research, education or administrative activities [3]. Productivity assessment helps in identifying highly productive faculty members, determining areas for faculty and departmental improvement, [4] and applying promotion and tenure processes [5]. Academic medical departments also use productivity assessment strategies along with reward schemes to incentivize targeted activities aligned with the organization’s mission and to increase efficiency [6]. These strategies typically cover clinical, research, education and administrative productivity. As a result this will enhance the overall revenue for the academic center as well as improve clinical teaching and training.

Multiple studies have evaluated the effects of compensating academic faculty based on their clinical, research, teaching as well as administrative performance [6–10]. A recent systematic review found that the introduction of productivity assessment strategies in academic medical centers improves research productivity, may improve clinical productivity, but has no effect on teaching productivity [3]. It also showed that compensation increases at both the group and individual levels, particularly for junior faculty.

While productivity based compensation may benefit academic departments and their faculty, it is known the extent to which they are being employed. The objective of this study was to evaluate whether and how academic Departments of Medicine in the United States measure faculty productivity for the purpose of salary compensation.

## Methods

### Study population

The study population consisted of the chairs of all academic Departments of Medicine in the United States. We excluded Departments of Medicine based in the Veterans Affairs Healthcare System, given that they have a specific compensation system already. We obtained the names and contact addresses of potential participants from a commercial vendor (Data Services, Inc.). The Institutional Review Board of the University at Buffalo approved the study protocol. All participants received a study information sheet that included all information typically included in a consent form.

### Survey questionnaire

We developed a brief, self-administered questionnaire about strategies used to measure the productivity of faculty for the purpose of salary compensation (Additional file 1). It included eight questions with 23 tabulated close-ended items. Four questions addressed the different types of faculty productivity: clinical, research, teaching and administrative. Each question started with whether the type of productivity was measured for the purpose of salary compensation. Positive answers led to further inquiries on whether productivity is converted into a standard unit of measurement, what exactly is measured, and how it is monetarily compensated. For completeness, the questionnaire included for each question an open answer (“other” category). We pilot tested the questionnaire with three administrative individuals at our institution.

### Data collection

Initially, letters were sent by one of the investigators (ABC) to the Chairs of Internal Medicine to inform them about the upcoming survey. Then, we mailed each participant a survey package that included: 1) a personalized cover letter explaining the purpose of the study, and signed by the Chair of the Department of Medicine at the University at Buffalo (ABC) and by the principal investigator (EAA); 2) a copy of the questionnaire; 3) a pre-addressed stamped return envelope; and 4) a monetary incentive in the form of a $5 Starbucks gift card for the chair’s administrative assistant (half of the participants were randomly selected to receive the card). We then sent a reminder letter by mail for all non-responders 2 weeks after the initial mailing, followed by a phone call reminder 2 weeks later. We used a tracking number for each questionnaire to avoid unnecessary reminders and allow re-contact of respondents who were agreeable to making their strategy available to other departments. We discarded the list of tracking numbers at the end of the study.

We attempted to maximize the response rate by using the following strategies proven to increase response rates to postal surveys, especially in physicians [11, 12]: monetary incentives for administrative assistants, university sponsorship (university logo on envelope and invitation letter), pre-mailing notification, personalized cover letter, colored ink, stamped return envelope, first class mailing, follow up mail and phone call. We kept the questionnaire relatively short and focused on factual questions.

### Statistical analysis

We conducted a descriptive analysis of the different methods of faculty productivity assessment used by the Departments of Medicine nationwide. We used Microsoft Office Access for data entry and management and SPSS version 13.0 (SPSS, Inc., Chicago, Illinois) for all analyses.

## Results

**Figure 1:**
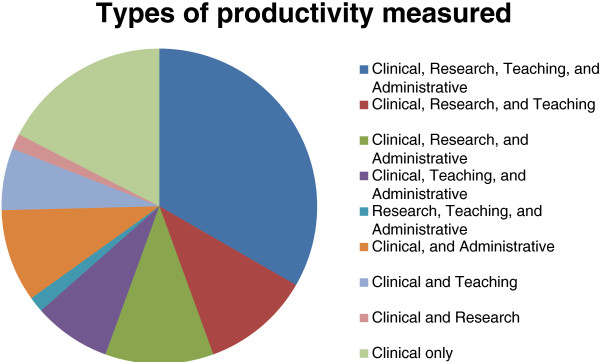
**Distribution of departments by the combination of the different types of productivity measured simultaneously.**

Chairs of 78 out of 152 eligible departments participated in the survey (51% response rate). The median number of full time faculty in departments of responding chairs was 130. Overall, 64 (82%) respondents reported measuring at least one type of faculty productivity for the purpose of salary compensation. The distribution of the different types of productivity was: 98% clinical, 61% research, 62% teaching, and 64% administrative (Figure 1).

Eighty four percent of the 63 respondents who reported measuring clinical productivity use a standardized unit including relative value units (RVUs), Association of American Medical Colleges (AAMC) RVUs, Work RVUs, Medical Group Management Association (MGMA) RVUs. The majority of departments used billable services (95%) and contractual services (70%) for measuring clinical productivity. Table 1 describes the clinical services that are measured and how they are monetarily compensated.

**Table 1:** **Description of measured clinical services and how they are measured amongst the 63 departments assessing clinical productivity (more than one option applies)**

What is measured		How it is monetarily compensated	
Billable services (e.g. patient encounters)	60/63(95%)	Compensated using fixed percentage of billable services	9/60 (15%)
		Compensated using Incremental amount after meeting a minimum	29/60 (48%)
		Other mode of compensation	15/60 (25%)
Contractual services (e.g. covering a clinical service)	44/63(70%)	Compensated using fixed percentage of related revenue	14/44 (32%)
		Compensated using incremental amount after meeting a minimum standard	6/44 (14%)
		Other modes of compensation	18/44 (41%)
Performance improvement	30/63(48%)	Clinical quality (e.g., HbA1c)	19/30 (63%)
		Utilization (e.g., length of stay)	13/30 (43%)
		Patient satisfaction	18/30 (60%)

Ninety five percent of 39 respondents who reported measuring research productivity use a standardized unit of measurement, mainly RVUs and grant money. The majority of departments measure research grants (87%), followed by career awards (33%) and full-length peer reviewed articles (31%). Table 2 describes the research activities that are measured and how they are monetarily compensated.

**Table 2:** **Description of measured research activities and how they are measured amongst the 39 departments assessing research productivity (more than one option applies)**

What is measured		How it is monetarily compensated	
Full length peer-reviewed publications	12/39 (31%)	Compensated using fixed amount for each publication	3/12 (25%)
		Compensated using incremental amount after meeting a minimum number	1/12 (8%)
		Compensated using other measures	7/12 (58%)
Career award, external recognition	13/39 (33%)	Compensated using fixed amount for each award	4/13 (31%)
		Compensated using other measures	9/13 (69%)
Research grants awarded	34/39 (87%)	Compensated using a fixed percentage of the monetary value of the grant	10/34 (29%)
		Compensated using fixed amount for each grant	3/34 (9%)
		Compensated using other measures	21/34 (62%)

Thirty percent of the 40 respondents who reported measuring teaching productivity use a standardized unit, mainly teaching RVUs. Table 3 describes the teaching activities that are measured and how they are monetarily compensated.

**Table 3:** **Description of measured teaching activities and how they are measured amongst the 40 departments assessing teaching productivity (more than one option applies)**

What is measured		How it is monetarily compensated	
Amount of clinical teaching (e.g., on the wards)	29/40 (72%)	Compensated using incremental amount after meeting a minimum	9/29 (31%)
		Compensated using other measures	13/29 (45%)
Amount of non-clinical teaching (e.g. lecture to medical students)	26/40 (65%)	Compensated using incremental amount after meeting a minimum	7/26 (27%)
		Compensated using other measures	17/26 (65%)

Only fifteen percent of the 41 respondents who reported measuring administrative productivity use a standardized unit of measurement, mainly based on percent effort and “meeting goals”. Table 4 describes the administrative activities that are measured and how they are monetarily compensated.

**Table 4:** **Description of measured administrative activities and how they are measured amongst the 41 departments assessing administrative productivity (more than one option applies)**

What is measured		How it is monetarily compensated	
Administrative responsibilities (e.g. division director)	39/41 (95%)	Compensated using fixed amount	30/39 (77%)
		Compensated using other measures	10/39 (26%)
Membership on committees (e.g., university, school, national)	12/41 (29%)	Compensated using fixed amount	6/12 (50%)
		Compensated using other measures	6/12 (50%)

Table 5 describes additional characteristics of compensation plans. The percentages of respondents who reported taking into account specific additional factors in their compensation plans were as follows: 34% for seniority, 77% for academic rank, and 14% for track (e.g., clinician-educator). According to respondents, compensation plans were in the form of a bonus on top of a fixed salary (66%) and/or an adjustment of salary based on previous period productivity (55%). A number of plans had a floor (a minimum) (47%) or a ceiling (a maximum) (31%). A total of 72% of compensation plans were reported to depend on the department financial performance.

**Table 5:** **Additional characteristics of compensation plan (N = 64)**

Compensation plan characteristic	n (%)
Takes into account seniority	22 (34)
Takes into account academic rank	49 (77)
Takes into account track (e.g., clinician-educator)	9 (14)
Is in the form of a bonus on top of a fixed salary	42 (66)
Is in the form of adjustment of salary based on previous period productivity	35 (55)
Has a floor (minimum)	30 (47)
Has a ceiling (maximum)	20 (31)
Depends on the department financial performance	46 (72)

## Discussion

We conducted a survey to evaluate whether and how academic Departments of Medicine in the United States measure faculty productivity for the purpose of salary compensation. We found that out of 78 responders, 82% reported measuring at least one type of productivity for the purpose of salary compensation, clinical productivity being included in almost all assessments (98%).

This study has a number of limitations. While the response rate of 51% compares favorably to previous surveys of health care professionals [13], it may lead to a potential for selection bias. If selection bias existed, it would have probably resulted in overestimating the percentage of departments measuring productivity for the purpose of salary compensation. On the other hand, the level of details collected by the questionnaire might be suboptimal for certain questions. For example, while 95% of departments measuring research productivity reported the use a standardized unit of measurement (mainly RVUs), we do not know how they actually converted measurements into the standardized unit. In terms of strengths, the questionnaire was simple and limited to eight questions. All questions were self-directed and ended with the option of an open-ended answer to allow answers not included as one of the question options.

Our survey showed almost all departments measure clinical productivity, and almost two-thirds measure research, academic and teaching productivity in a similar distribution (Figure 1). Most of the previous studies that measured performance-based compensation have focused mainly on clinical activity [10, 14–16]. Factors that are usually cited to improve clinical practice include mainly reimbursement, feedback relative to other physicians and threat of legal action [17]. Indeed, departments can implement performance-based reimbursement to improve physician’s clinical productivity. For example the Department of Internal Medicine at the University of Kansas developed a new metric system, the financial value unit or FVU, which analyzes clinical compensation with clinical work productivity. The implementation of this metric helped improve overall clinical productivity along with increasing physician compensation [18].

The fact that more than 80% of departments are assessing faculty productivity indicates a wide belief that such assessment is beneficial. At the same time, a substantive percentage of departments do not measure non-clinical types of productivity i.e., research, teaching or administrative. There is also a very wide variation in what exactly is measured, how it is measured and most importantly how it is compensated. This leads to the question about whether a unifying system can be helpful to increase overall productivity.

Abouleish has argued that an incentive program focused on specific goals – or types of productivity – is more effective than productivity-based compensation programs [6]. In one example, the anesthesiology department at the University of Pittsburgh developed an integrated academic and clinical compensation plan based on a merit system that resulted in an overall improvement of faculty productivity [19]. A unifying system should take into account all activities in the department, give relative values for each activity, and convert each value to a common measure [20, 21]. As a result the system may become overly complex [19]. On the other hand, it might be difficult or challenging to measure teaching productivity, [22] and even certain clinical activities [23].

The findings have also implications for future research. Specifically, there is a need for good quality research (e.g., large controlled observational or before-after studies with careful handling of confounding) about the benefits and harms of productivity assessment strategies. In addition, a central repository would assist researchers in designing their evaluation studies.

## Conclusions

In a survey of Chairs of academic Departments of Medicine in the United States, we found that most of these departments measure faculty productivity and convert it into standardized units for the purpose of salary compensation. While the vast majority measure clinical productivity, about two thirds measure the other types. The exact activities measured and how they are monetarily compensated varied widely between departments. On one hand, this might indicate that departments are adapting the way they measure faculty productivity to their local circumstances, rules and regulations. On the other hand, it suggests that departments would benefit from sharing experiences in terms of what worked and what didn’t. Ultimately, it might help to have standardized and validated measures for the different types of productivity that could be used across departments.

## Electronic supplementary material

Additional file 1: **Survey questionnaire.** (PDF 120 KB)

## Abbreviations

RVUs: Relative value units.
