# Supplementary material for: Assessment of faculty productivity in academic departments of medicine in the United States: a national survey
Source: BMC Med Educ. 2014 Sep 26;14:205. doi: 10.1186/1472-6920-14-205 (PMC4189191; doi:10.1186/1472-6920-14-205)
Supplement: Supplementary file 1 — Additional file 1: Survey questionnaire. (PDF 120 KB) [file 12909_2014_1031_MOESM1_ESM.pdf]

1) Please provide the number of full time faculty in your department: N=

2) Do you measure productivity of **faculty** for the purpose of **salary compensation**?
   □ No → stop here
   □ Yes → please answer the remaining questions

3) Do you measure **clinical productivity** for the purpose of salary compensation?

□ **No**	□ **Yes** (please fill out the table)	
	**Is it converted into a standardized unit of measurement?**<br>□ No □ Yes, please specify (e.g., MGMA RVUs, AAMC RVUs, other scale):	
	**What is measured:**	**How it is monetarily compensated:**
	□ Billable services<br>(e.g. patient encounters)	□ Fixed percentage of billable services<br>□ Incremental amount after meeting a minimum<br>□ Other:
	□ Contractual services<br>(e.g. covering a clinical service)	□ Fixed percentage of related revenue<br>□ Incremental amount after meeting a minimum standard<br>□ Other:
	□ Performance improvement<br>(check all that apply)<br>□ Clinical quality (e.g., HbA1c)<br>□ Utilization (e.g., length of stay)<br>□ Patient satisfaction	□ Fixed amount<br>□ Incremental amount after meeting a minimum standard<br>□ Other:
	□ Other:	□ Specify:

4) Do you measure **research productivity** for the purpose of salary compensation?

□ **No**	□ **Yes** (please fill out the table)	
	**Is it converted into a standardized unit of measurement?**<br>□ No □ Yes, specify (e.g., RVUs, other scale):	
	**What is measured:**	**How it is monetarily compensated:**
	□ Full length peer-reviewed publications	□ Fixed amount for each publication<br>□ Incremental amount after meeting a minimum number<br>□ Other:
	□ Career award, external recognition	□ Fixed amount for each award<br>□ Other:
	□ Research grants awarded	□ Fixed percentage of the monetary value of the grant<br>□ Fixed amount for each grant<br>□ Other:
	Are federal grants valued higher than other grants?	□ Yes □ No
	□ Other:	□ Specify:

5) Do you measure **teaching/educational productivity** for the purpose of salary compensation?

□ **No**	□ **Yes** (please fill out the table)	
	**Is it converted into a standardized unit of measurement?**<br>□ No □ Yes, specify (e.g., RVUs, other scale):	
	**What is measured:**	**How it is monetarily compensated:**
	□ Amount of clinical teaching<br>(e.g., on the wards)	□ Incremental amount after meeting a minimum<br>□ Other:
	□ Amount of non clinical teaching<br>(e.g. lecture to medical students)	□ Incremental amount after meeting a minimum<br>□ Other:
	□ Other:	□ Specify:

6) Do you measure **administrative productivity** for the purpose of salary compensation?

□ **No**	□ **Yes** (please fill out the table)	
	**Is it converted into a standardized unit of measurement?**<br>□ No □ Yes, specify (e.g., RVUs, other scale):	
	**What is measured:**	**How it is monetarily compensated:**
	□ Administrative responsibilities<br>(e.g. division director)	□ Fixed amount<br>□ Other:
	□ Membership on committees<br>(e.g., university, school, national)	□ Fixed amount<br>□ Other:
	□ Other:	□ Specify:

7) Check all that apply about the compensation plan:

Takes into account:	□ Seniority □ Academic rank		□ Track (e.g., clinician-educator)<br>□ Other:
Is in the form of:	□ Bonus on top of a fixed salary<br>□ Adjustment of salary based on previous period productivity<br>□ Other:		
Has:	□ A floor (minimum)		□ A ceiling (maximum)
Depends on the department financial performance:	□ Yes		□ No

Comments:

8) Are you willing to share you compensation plan with other departments?

□ No □ Yes (please provide contact details):
